# Propolis changes the anticancer activity of temozolomide in U87MG human glioblastoma cell line

**DOI:** 10.1186/1472-6882-13-50

**Published:** 2013-02-27

**Authors:** Renata Markiewicz-Żukowska, Maria H Borawska, Anna Fiedorowicz, Sylwia K Naliwajko, Diana Sawicka, Halina Car

**Affiliations:** 1 The Department of Bromatology, Medical University of Bialystok, Mickiewicza 2D, 15-222, Bialystok, Poland; 2 The Department of Experimental Pharmacology, Medical University of Bialystok, Szpitalna 37, 15-295, Bialystok, Poland; 3 The Center of Experimental Medicine, Medical University of Bialystok, Marii Sklodowskiej-Curie 24A, 15-276, Bialystok, Poland

**Keywords:** Propolis, Glioblastoma, Temozolomide, Cell viability, DNA synthesis, NF-κB

## Abstract

**Background:**

Propolis is a honey bee product which contains many active compounds, such as CAPE or chrysin, and has many beneficial activities. Recently, its anti-tumor properties have been discussed. We have tested whether the ethanolic extract of propolis (EEP) interferes with temozolomide (TMZ) to inhibit U87MG cell line growth.

**Methods:**

The U87MG glioblastoma cell line was exposed to TMZ (10-100 μM), EEP (10-100 μg/ml) or a mixture of TMZ and EEP during 24, 48 or 72 hours. The cell division was examined by the H^3^-thymidine incorporation, while the western blot method was used for detection of p65 subunit of NF-κB and ELISA test to measure the concentration of its p50 subunit in the nucleus.

**Results:**

We have found that both, TMZ and EEP administrated alone, had a dose- and time-dependent inhibitory effect on the U87MG cell line growth, which was manifested by gradual reduction of cell viability and alterations in proliferation rate. The anti-tumor effect of TMZ (20 μM) was enhanced by EEP, which was especially well observed after a short time of exposition, where simultaneous usage of TMZ and EEP resulted in a higher degree of growth inhibition than each biological factor used separately. In addition, cells treated with TMZ presented no changes in NF-κB activity in prolonged time of treatment and EEP only slightly reduced the nuclear translocation of this transcription factor. In turn, the combined incubation with TMZ and EEP led to an approximately double reduction of NF-κB nuclear localization.

**Conclusions:**

We conclude that EEP presents cytotoxic properties and may cooperate with TMZ synergistically enhancing its growth inhibiting activity against glioblastoma U87MG cell line. This phenomenon may be at least partially mediated by a reduced activity of NF-κB.

## Background

Glioblastoma is a common primary brain tumor which demonstrates a high proliferation rate and an aggressive growth pattern and is largely resistant to chemotherapy [1]. One of the most promising drugs for brain tumor therapy is temozolomide (TMZ) (8-carbamoyl-3-methylimidazo [5,1-*d*]- 1,2,3,5-tetrazin-4 (3H)-one), an oral alkylating agent belonging to imidazotertrazines [2], which exhibits anti-cancer properties against malignancies [1,3,4]. According to Uzzaman *et al,*[5] the average survival of patients with glioblastoma after TMZ treatment is 22 months, which is still not enough to be satisfied with the therapy.

Propolis is composed of flavonoids, fatty, aliphatic and aromatic acids, steroids, aminoacids and vitamins, such as B_1_, B_2_, E, C, flavonoids, alcohols, terpenes, sugars and esters [6,7]. A natural honeybee product, propolis has been demonstrated in many studies due to its antibacterial, antifungal [8-10], antiviral [11] hepatoprotective and immunostimulative activities [12]. It has been showed that the efficacy of propolis depends on the contents of the mentioned ingredients in particular flavonoids [13,14].

The anti-cancer activities of propolis have been presented in various culture cell lines, such as mammary carcinoma (MCA), human epithelial carcinoma (HeLa), human leukemia (HL-60, CI41, U937), human ovarian carcinoma (SK-OV-3), human lung carcinoma (NCI-H358), human hepatocellular carcinoma (HepG2), human cervical cancer (ME180) and human pancreatic cancer (PANC-1, BxPC-3) cells [15-19]. The anti-tumor activities of propolis were exerted by proliferation arrest and/or induction of apoptosis in a variety of cancer cells.

One of the benefits of anticancer therapy is the induction of apoptosis by the kinases activation and the release of cytochrome c into the cytosol, which activates caspases [15,16]. This causes the cell cycle arrest by the suppression of complexes of cyclins and cyclin-dependent protein kinases in cancer cells [17-19].

A recent study showed that TMZ administered together with propolis [20] enhanced the sensitivity of human brain cancer cells, indicating that the combination of TMZ with natural products may be more effective in glioma therapy than using TMZ alone. It seems that naturally bioactive compounds may have a co-operative action with chemotherapeutics. The combination of TMZ and natural anti-cancer agents is suggested as a new approach in Central Nervous System (CNS) tumor treatment.

The aim of our study was to examine the influence of EEP on TMZ anti-cancer activity in U87MG human glioblastoma cell line, expressed as changes in cell viability and proliferation. We have also established a correlation between cell growth inhibition by TMZ, EEP and their combination and nuclear factor type B (NF-κB) activity.

## Methods

### Reagents

TMZ, thiazolyl blue tetrazolium bromide (MTT) and dimethyl sulfoxide (DMSO) were obtained from Sigma-Aldrich (St. Louis, MO, USA). Propolis of *Apis mellifera* was collected in the Podlasie region (the north-west part of Poland) in August 2010. To obtain ethanolic extract, propolis was crushed and 20 g were extracted in a shaker with 80 g of 95% ethanol for 6 h in a darkened place. The extract was filtered, concentrated and lyophilized. The dry extract was protected from light and kept frozen at −20°C. The yield of the prepared extract (% w/w), in terms of the starting material, was 16.3. The extract was dissolved in DMSO and prepared as 1 mg/ml stock solution in medium.

### Cell culture

The studies were performed on a human glioblastoma cell line U87MG (HTB-14) purchased from American Type Culture Collection, (Rockville, MD). The cells were maintained in Eagle's Minimal Essential Medium Eagle with l-glutamine (292 mg/L) (PAA Laboratories GmbH, Pasching, Austria) supplemented with 10% fetal bovine serum (PAA Laboratories GmbH, Pasching, Austria) without antibiotics in a humidified incubator at 37°C and 5% CO_2_ atmosphere. Sub-confluent cells were detached with Trypsin-EDTA solution (PAA Laboratories GmbH, Pasching, Austria) in calcium-free phosphate buffered saline (PBS) (Biomed, Lublin, Poland) and counted in hemocytometers.

### Cytotoxicity assay

The effects of TMZ (10, 20, 50, 100 μM), EEP (10, 20, 30, 50, 100 μg/ml) and EEP combined with TMZ (20 μM) on the viability of glioblastoma cell line (U87MG) were studied after 24 h, 48 h and 72 h of treatment. Cells were seeded into 96-well plates in a volume of 200 μl per well at density of 2 × 104 cells/well and grown for 22 h at 37°C in a humidified 5% CO_2_ incubator. Cell viability was measured by a quantitative colorimetric assay using MTT [21]. The data was expressed as a percentage of control.

### H^3^-thymidine incorporation

U87MG cells were plated in 24-well plates and exposed to a medium containing DMSO (control), TMZ, EEP or EEP with TMZ. Cells were cultured 20, 44 and 68 hours prior to the addition of 0.5 μCi of H^3^-thymidine per well. After 4 hours of incubation, the medium was removed and cells were washed twice with cold 0.05 M Tris-HCl and 5% trichloroacetic acid, scrapped and transferred to a scintillation cocktail. The level of incorporated H^3^-thymidine was assessed using Beckman liquid scintillation counter.

### Western blot

Scrapped cells were centrifuged and frozen in -80°C until use. The nuclear proteins were extracted using Nuclear and Cytoplasmic Extraction Reagents (NE-PER) kits (Thermoscientific) according to the manual provided by the manufacturer. 50 μg of protein from each sample was separated by 12% sodium dodecyl sulfate polyacrylamide gel electrophoresis (SDS-PAGE) and immobilized onto the PVDF membrane (Millipore). The immunoblots were incubated with primary antibodies, at a dilution of 1:500 for p65 (Cell Signaling Technology) and 1:1000 for H3 (histone H3) (Sigma-Aldrich) overnight and then with the secondary antibodies conjugated with alkaline phosphatase (AP) (Cell Signaling Technology, Sigma–Aldrich, respectively). Immunoreaction was developed with nitro blue tetrazolium- 5-Bromo-4-chloro-3-indolyl phosphate (NBT-BCIP) system. The optical density of the bands was established using INTAS device. H3 levels were considered as an internal control of the amount of loaded protein. The results were normalized and presented as a percentage of control. The results were calculated from five independent experiments.

### Enzyme-linked immunosorbent assay (ELISA)

Nuclear extracts in amount of 40 μg per well, utilized previously in Western blotting (as described above) were used in ELISA. The experiments were performed using DNA-binding ELISAs for activated NF-κB transcription factors (TransAM) NFκB p50 (Active Motif) in accordance with the instructions of manufacturer. The results are shown as a percentage of the control value and are calculated from three independent experiments.

### Statistical analysis

The data was expressed as a mean value of percent of control ± standard deviation. All data was analyzed using STATISTICA, Version 9.0 using the Student-t test to calculate the value significance. P values < 0.05 were accepted as statistically significant.

## Results

### The effect of TMZ on the viability of glioblastoma cell line (U87MG)

**Figure 1:**
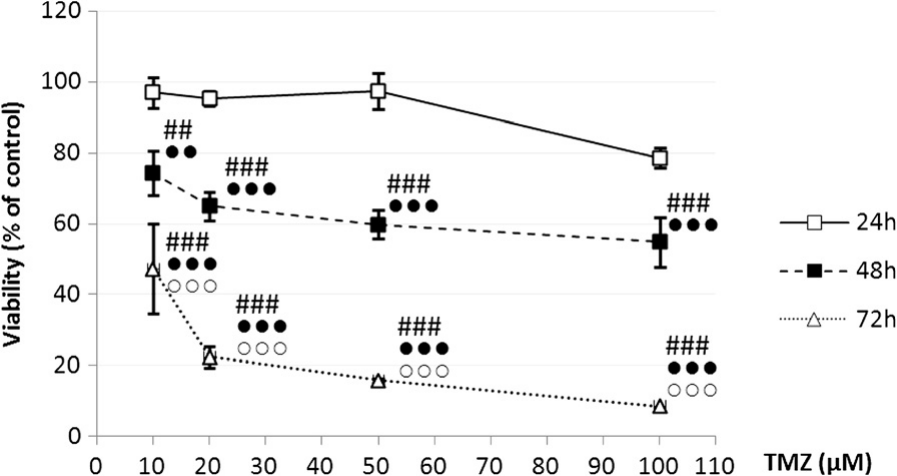
**Viability of U87MG (% of control) after incubation with temozolomide.** The results are presented as a percentage of control. ## p < 0.01, ### p < 0.001 vs. control; ● ● p < 0.01, ● ● ● p < 0.001 vs. equivalent of 24 h groups;○○○ p < 0.001 vs. equivalent of 48 h groups.

After 24 h of incubation, TMZ at concentrations 10 μM-50 μM did not significantly alter the viability of the U87MG cell line (Figure 1). The marked reduction of viable cell number (79% of control) at that time point was observed only when TMZ was used in concentration of 100 μM. After 48 h of exposure, the viability of glioma cells decreased gradually with increasing TMZ concentrations reaching 74.40% of control for the lowest concentration and 54.90% for the highest. A strong reduction in the number of living cells in relation to control (from 47.30% to 8.60%) was reported after 72 h of treatment with TMZ for all TMZ concentrations used. The effect of cell growth inhibition by TMZ potentiated with time of exposure for each amount of the drug.

### The effects of *EEP* and *EEP* with TMZ on the viability of glioblastoma cell line (U87MG)

Basing on the comparative analysis of the effects of different TMZ concentrations on U87MG cell growth, we established that 20 μM of TMZ is the most appropriate concentration which should be used in further experiments (Figure 1). Additionally, we selected the concentration close to the content of TMZ in brains of patients with gliomas. It was shown, using a microdialysis study, that the peak concentration of TMZ in brain interstitium was about 0.6 μg/ml (3 μM) [22] and TMZ concentrations in a normal brain calculated from pharmacokinetic model ranged from 1.8 to 3.7 μg/ml (9 μM – 20 μM) [23].

**Figure 2:**
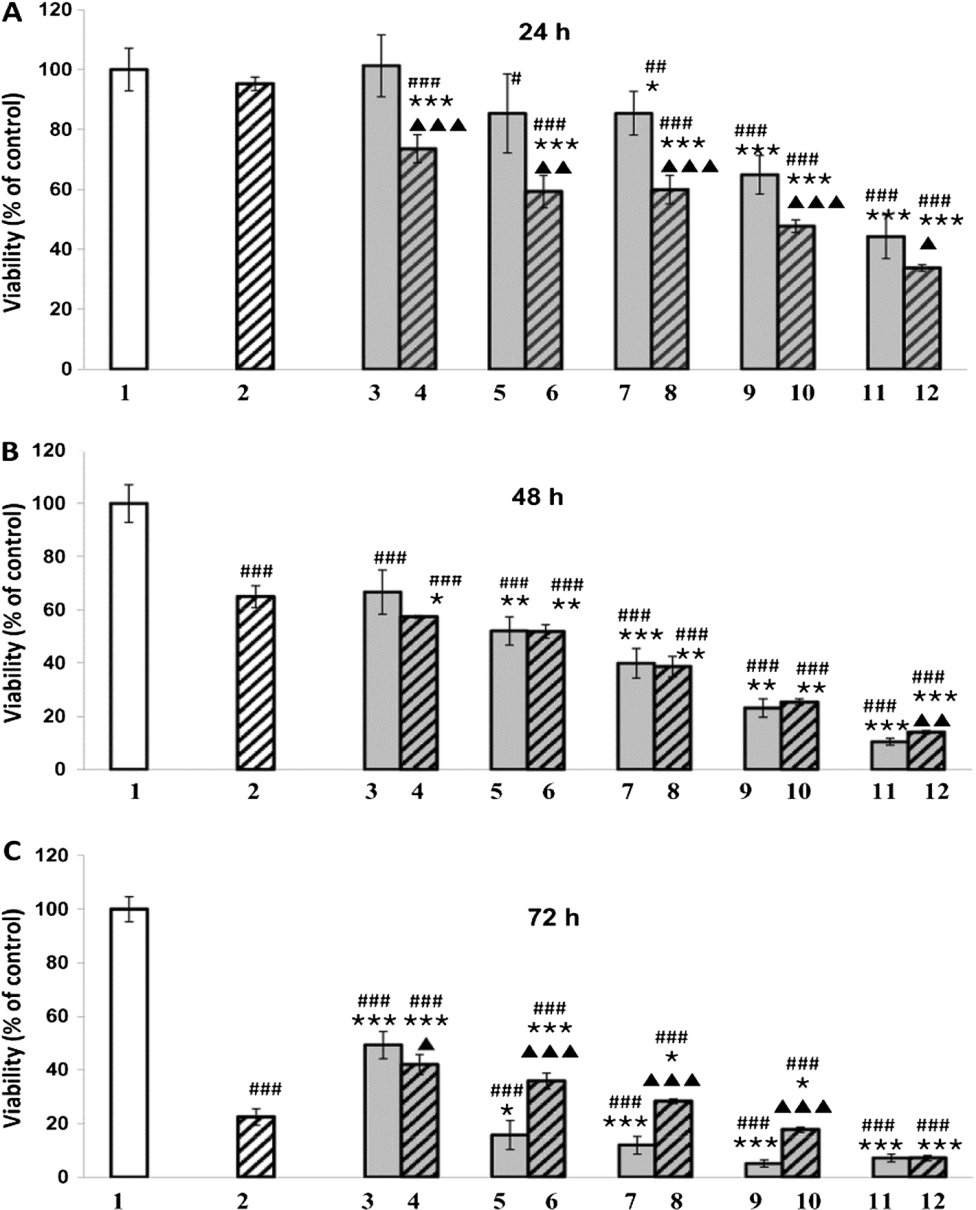
**Viability of U87MG (% of control) after incubation with EEP and temozolomide.** Control – (1), Temozolomide (2 - TMZ 20 μM), EEP (3 - 10 μg/ml, 5 - 20 μg/ml, 7 - 30 μg/ml, 9 - 50 μg/ml, 11 - 100 μg/ml), EEP administered together with temozolomide (4 – EEP 10 μg/ml + TMZ 20 μM, 6 - EEP 20 μg/ml + TMZ 20 μM, 8 - EEP 30 μg/ml + TMZ 20 μM, 10 - EEP 50 μg/ml + TMZ 20 μM, 12 - EEP 100 μg/ml + TMZ 20 μM). Viability of U87MG cells after 24 (**A**), 48 (**B**) and 72 (**C**) hours of incubation. The results are presented as a percentage of control. **A**/^#^p < 0.05, ^##^p < 0.01, ^###^p < 0.001, vs. control; *p < 0.05, ***p < 0.001, vs. TMZ; ^▲ ^p < 0.05, ^▲ ▲ ^p < 0.01, ^▲ ▲ ▲ ^p < 0.001, vs. EEP; **B**/ ^###^p < 0.001, vs. control; *p < 0.05; **p < 0.01; ***p < 0.001, vs. TMZ; ^▲ ▲ ^p < 0.01, vs. EEP; **C**/^###^p < 0.001, vs. control; *p < 0.05, ***p < 0.001, vs. TMZ; ^▲ ^p < 0.05, ^▲ ▲ ▲ ^p < 0.001, vs. EEP. All statistical analysis were performed using Student-t test.

We found that EEP induces significant dose- and time-dependent reductions of cell viability in comparison with control (Figure 2). Interestingly, EEP was a stronger inducer of U87MG cell growth arrest than TMZ, starting from the concentration of 30 μg/ml for 24 hours and 20 μg/ml for 48 and 72 hours of exposition (Figure 2).

The combined administration of TMZ at a concentration of 20 μM and EEP at various concentrations brought diverse results. Clear, synergistic action of EEP and TMZ was noted for 24-hour treatment. The cell viability was greater for all concentrations of separately-administrated EEP (from about 100% to 44%) and 20 μM TMZ (about 95%) in comparison with the combined administration of 20 μM TMZ and a corresponding dose of EEP (from about 74% to 34%, respectively from the lowest to the highest concentration of EEP) (Figure 2A). We have noted that 48 hours after exposure, a reduction of cell viability by a combined administration of EEP and TMZ remained on a similar level as that revealed by EEP alone (from about 67% to 10% for propolis and from 57% to 14% of control for TMZ with EEP, respectively) and in all cases was higher than the effect of TMZ (20 μM) alone (65% of control) (Figure 2B). 72 hours after exposure, the result of EEP action on U87MG treated simultaneously with TMZ was dependent on the concentration of EEP. Its lower concentrations (10-30 μg/ml) exerted a lesser effect on the reduction in the number of living cell treated at the same time with TMZ than TMZ alone (from about 42% to 28% of control for TMZ with EEP, respectively and about 22% of control for TMZ alone). In contrast, 50 and 100 μg/ml of EEP were able to inhibit TMZ-treated cell growth stronger than TMZ alone (about 18% and 7% of control, respectively). However, the use of a combined administration of TMZ and EEP led to a better cell viability for 20-50 μg/ml of EEP in comparison with propolis used at those concentrations alone (36% to 18% of control for TMZ with EEP and 16% to 5% of control for EEP alone). Only at the concentration of 10 μg/ml EEP demonstrate a higher inhibition of the viability in combination with TMZ (42% of control) than alone (49% of control). For 100 μg/ml of EEP both types of treatments showed similar effects (about 7% of control for TMZ and EEP and 7% of control for EEP alone) (Figure 2C).

### The effect of *EEP* and *EEP* with TMZ on H^3^-thymidine incorporation in the glioblastoma cell line (U87MG)

**Figure 3:**
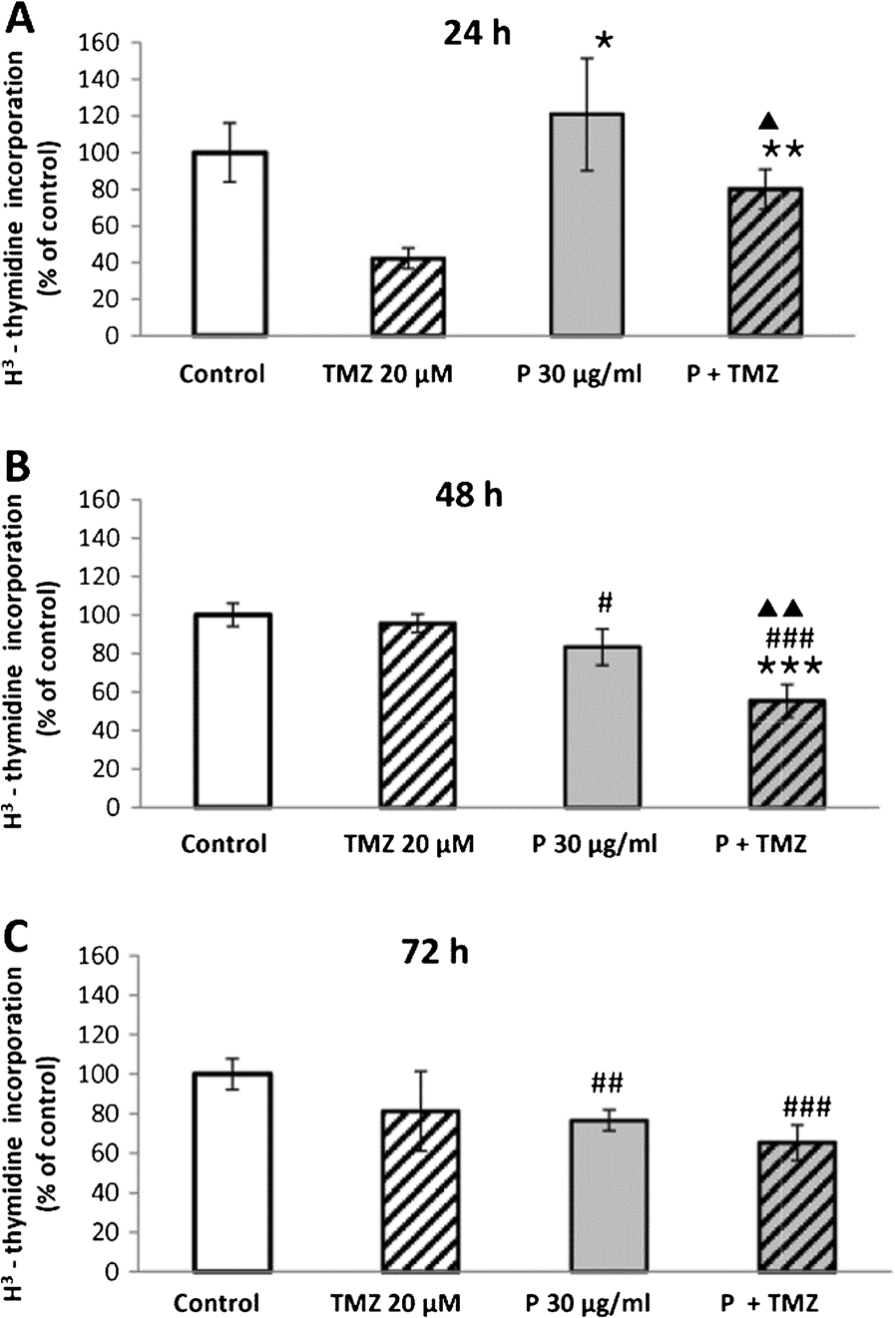
**The effects of EEP or/with temozolomide on H**^**3**^**- thymidine incorporation in U87MG cells.** EEP (P 30 μg/ml), temozolomide (TMZ 20 μM), EEP administered together with temozolomide (P+TMZ). H^3^- thymidine incorporation after 24 (**A**), 48 (**B**) and 72 (**C**) hours of incubation. The results are presented as a percentage of control. Significant changes obtained from the Student-t test are indicated at the columns with: **A**/ ^##^p < 0.01, vs. control; *p < 0.05, **p < 0.01, vs. TMZ; ^▲ ^p < 0.05, vs. EEP; **B**/ ^#^p < 0.05,^###^p < 0.001, vs. control; ***p< 0.001, vs. TMZ; ^▲ ▲ ^p< 0.01, vs. EEP; **C**/ ^##^p < 0.01; ^###^p < 0.001, vs. control.

TMZ revealed a strong inhibitory potential to U87MG cells after a 24-hour exposition leading to an almost 60% decrease of H^3^-thymidine incorporation versus control (Figure 3A). At the same time, EEP exhibited a minor ability to enhance proliferation and a combination of TMZ and EEP was less powerful to arrest cell division (about 80% of control) than TMZ alone (about 40% of control). 48-hour exposition did not cause a reduction of proliferation rate of TMZ treated cells, whereas EEP lowered DNA synthesis by approximately 20% in relation to control. Moreover, a combination of EEP with TMZ exhibited the synergistic effect decreasing proliferation by about 50% (Figure 3B). After 72 hours of exposure, the treatment of U87MG cells with TMZ alone did not alter H^3^-thymidine incorporation in comparison with control. In turn, EEP used alone caused about 20% reduction in cell division versus control and a combined treatment of EEP and TMZ led to about 30% of proliferation decline (Figure 3C).

### The effect of *EEP* and *EEP* with TMZ on NF-κB activity in glioblastoma cell line (U87MG)

**Figure 4:**
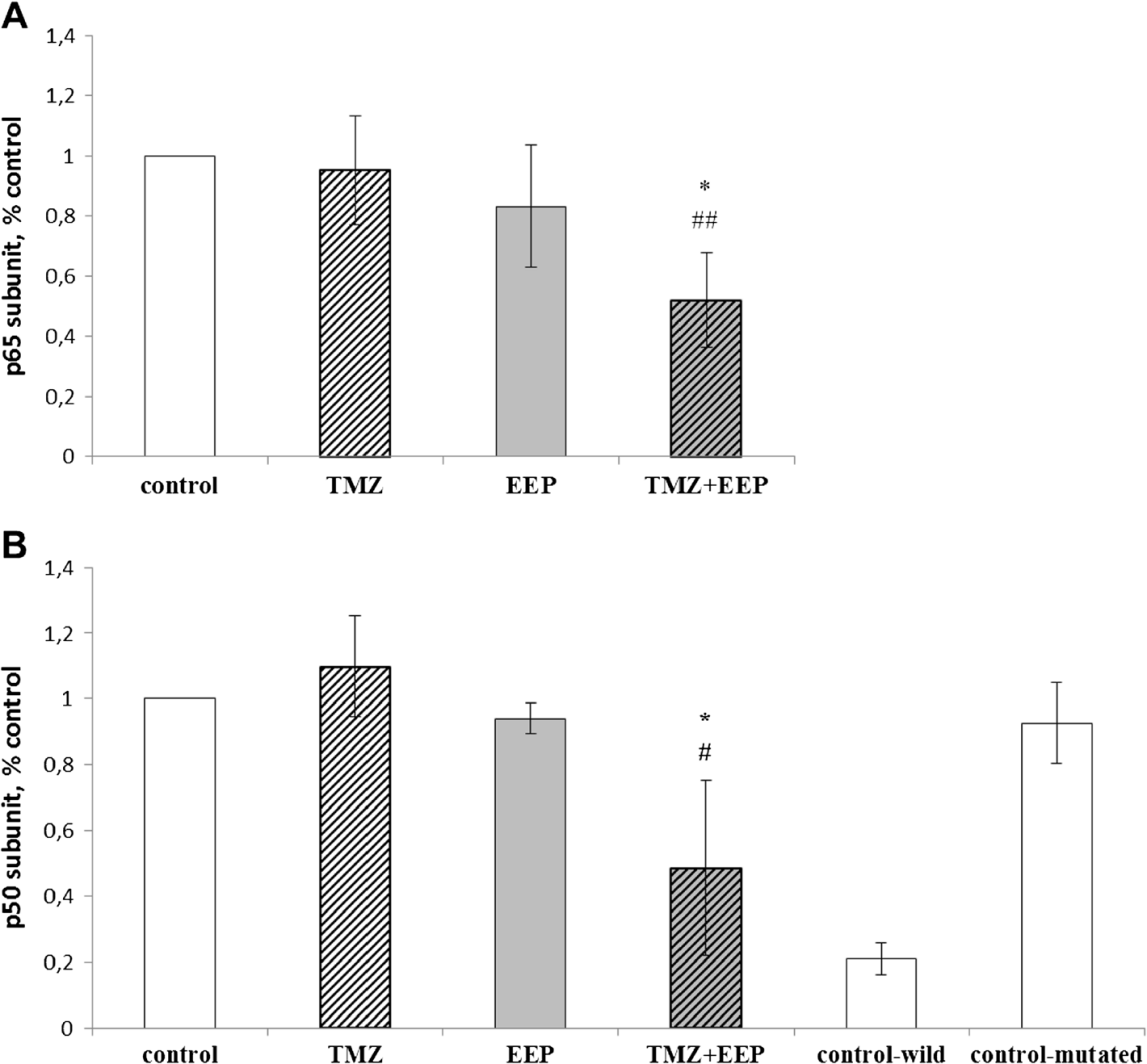
**The effects of EEP or/with temozolomide on NFκB activity in U87MG cells.** EEP (30 μg/ml), temozolomide (TMZ 20 μM), EEP administered together with temozolomide (TMZ + EEP). NFκB activity: **A**/ protein p65 based on the Western blot analysis, **B**/ protein p50 based on the Elisa method. The results are presented as a percentage of control. Significant changes obtained from the Student-t test are indicated at the columns with: **A** and **B**/ ^##^p < 0.01, vs. control; *p < 0.05, vs. TMZ.

We examined whether there is a correlation between a reduced viability and proliferation of the glioblastoma cells and the activity of NF-κB. The U87MG cells were incubated in medium containing TMZ (20 μM) or EEP (30 μg/ml) alone or a combination of TMZ and EEP (20 μM and 30 μg/ml, respectively) during 72 hours. Using the western blot method for detection of p65 subunit of NF-κB (Figure 4A) and ELISA test to measure the concentration of its p50 subunit (Figure 4B) in the nucleus, we noted a perfect correlation of results obtained for both subunits. We found that TMZ did not alter the NF-κB activity in U87MG cells since nuclear localization of both p65 and p50 remained at the level similar to control. Moreover, EEP alone led to only a slight decrease of nuclear content of both subunits. On the other hand, the incubation of cells with mixture of TMZ and EEP resulted approximately in 50% reduction of NF-κB activity, which clearly suggests a synergic effect of EEP and TMZ. To verify the ELISA specificity, we performed the pre-incubation of the control nuclear extracts with wild-type or mutated consensus sequence for NF-κB. The test of the competition showed a dramatic decline of p50 signal when a wild-type sequence was used and only a minor decrease using a mutated sequence.

## Discussion

In this work we have presented the cooperative effect of temozolomide and ethanolic extract of propolis on the growth of glioblastoma U87MG cell line. We examined the ethanolic extract of propolis because it is a common dietary supplement. Patients during chemotherapy may be tempted to self-medicate with natural supplements. There is data indicating that some dietary supplements may impair the activity of anticancer drugs. Therefore, the simultaneous intake of chemotherapeutics and folk medicine products by oncogenic patients might lead to the worsening the effects of therapy [24]. This kind of extract is commonly used in the cell cultured (glioblastoma, neuronal cells) experiments [20,25,26]. The main idea of our study was to verify whether propolis, a widely-consumed natural product, interferes with TMZ, a drug approved in glioma therapy. TMZ acts on cells in the cell cycle stage independent manner [2], producing DNA lesions and the methyl guanine (O6-MG) adduct is considered to play an essential role in TMZ cytotoxicity [27]. The main enzyme responsible for glioblastoma multiform (GBM) resistance to TMZ is O6-MG-DNA methyltransferase (MGMT), which takes off the methyl adducts from the DNA [15]. Although MGMT gene promoter is entirely methylated in U87MG cells and the protein remains on undetectable level [28], these cells reveal a partial resistance to TMZ.

We have found concentration- and time-dependent decrement in viability of U87MG cells treated both with TMZ and EEP. Similar results were obtained by Borges *et al*[20], who showed antiproliferative properties of Tubi-bee propolis in glioblastoma and fibroblast cell lines and its synergistic action with TMZ. An amount of evidence shows that propolis causes a reduction of cancer cell growth due to proliferation arrest and apoptosis. However, its effects are dependent on the cell type and the geographic region where propolis comes from [29]. The inhibition of DNA synthesis was reported for Turkish propolis, which reflected its anti-tumor influence [30]. Ethanolic extract of Brazilian propolis administrated to U937 lymphoma cells caused a reduced cell growth and inhibition of DNA, RNA and protein synthesis [31] and Brazilian red propolis eliminated 100% of PANC-1 cell line, when added to the cell medium [32]. The mechanism by which propolis supports TMZ anti-cancer activity is difficult to elucidate due to a great number of its components. Many active substances, such as quercetin, CAPE or chrysin [33], may be involved in a cytotoxic effect of propolis, and most of them have not been fully tested yet. The analysis of propolis obtained from Podlasie region revealed enrichment in CAPE and chrysin (manuscript under revision). These two compounds are known to exhibit particularly high biological activity. Interestingly, CAPE and chrysin bring a minor effect on cell growth when used separately than crude propolis [34]. This observation provides the basis for studies of the whole propolis extract rather than its individual ingredients, expecting that multi-factor activity may bring a stronger and more complex effect on cancer.

NF-κB is an essential survival factor for many glioblastomas including U87MG cell line [35]. Glioblastomas responded to NF-κB inhibition by reducing the growth rate and induction of apoptosis. Moreover, U87MG cells grafted into mice formed a tumor, which was reduced in size by 40% by blocking NF-κB signaling [36]. Propolis contains compounds, such as CAPE [16] or chrysin [37], which can inhibit this transcription factor activity. Taking into account the strong impact on NF-κB activation presented by propolis-derived compounds we verified whether propolis from Poland modulates the NF-κB translocation to the nucleus in U87MG cells exposed simultaneously to TMZ. NF-κB transcription factor is a dimmer, which may consist of subunit p65 (RelA), p50, p52, c-Rel and RelB, where the most common set is p65-p50. NF-κB is anchored in the cytoplasm in its inactive form by coupling to the inhibitory protein of nuclear factor type B (IκB). When activated, nuclear signal localization (NLS) of NF-κB unveils and the protein proceeds to nucleus, where it binds to the promotor regions of NF-κB-responsive genes [38]. In our studies we have found that EEP had only a modest effect on NF-κB nuclear localization in U87MB cells, which could be a consequence of too low concentrations of NF-κB-inhibiting compounds in the extract we used in our experiments. TMZ revealed no influence on the transcription factor activity. Interestingly, a combined administration of TMZ and EEP led to 50% reduction of NF-κB nuclear translocation. Chaturvedi *et al*[39] in their review mention that NF-κB overactivity is frequently found in different types of cancers and the reduction of its transcriptional action could serve as a treatment supplementary to chemotherapy. Indeed, it was shown that preventing the conditional activation of NF-κB inhibited melanoma development in mice [40].

To elucidate how TMZ and propolis cooperate to reduce NF-κB activation is a complex problem. The expression of NF-κB may be regulated basing on p53-dependent mechanism [41]. Interestingly, an important feature of most of gliomas is the lack of mutation in *p53* gene, which is also characteristic of U87MG cell line [42]. It was reported that the downregulation of p65 subunit in the squamous cell carcinoma of the head and neck cell lines required a presence of p53 protein [43]. Moreover, p53 augmented highly in U87 cells after exposure to TMZ [44]. Thus, a slight and not significant effect of propolis on the reduction of NF-κB activity may be potentiated by p53 stimulated by TMZ. It seems that Akt-NF-κB relation may also be important in gliomas. Although the epidermal growth factor receptor/ phosphatidylinositol-3 kinases/ protein Kinase B (EGFR/PI3K/Akt) pathway is not mutated in U87MG cell line [45], these cells contain phosphatase and tensin homolog (*pten)* deletion [46], an inhibitor of Akt-mediated signaling [47]. Zhang *et al*[48] showed that an activated EGFR/Akt pathway positively influenced NF-κB signaling in U87MG cells. This is especially important since TMZ can stimulate PI3K/Akt signal transduction [49]. However, it was also shown that TMZ induced increase of Akt activation is rather marginal [50] in U87MG cells, which may explain our results, where TMZ did not alter NF-κB activity. It is likely that propolis exerts an antagonistic effect on Akt signaling since its components were shown to inhibit both NF-κB activation and Akt phosphorylation [51,52]. Propolis may then interfere with the TMZ-dependent PI3K/Akt signaling leading to partial decrease of NF-κB activity.

## Conclusions

In this study we have shown that EEP collaborates with temozolomide promoting the inhibition of glioblastoma cell growth in comparison to these two agents used separately. The potentiation of anticancer activity of temozolomide by EEP may be mediated by synergistic influence of both agents on reduction of NF-κB activity.

## Abbreviations

AP: Alkaline phosphatase;BxPC-3: Human pancreatic cancer cell line;CAPE: Caffeic acid phenethyl ester;CI41: Human leukemia cell line;CNS: Central Nervous System;DMSO: Dimethyl sulfoxide;EEP: Ethanolic extract of propolis;EGFR: Epidermal growth factor receptor;ELISA: Enzyme-linked immunosorbent assay;EPC: Electronic pressure control;GBM: Glioblastoma multiform;HeLa: Human epithelial carcinoma cell line;HepG2: Human hepatocellular carcinoma cell line;HL-60: Human leukemia cell line;IκB: Inhibitory protein of nuclear factor type B;MCA: Mammary carcinoma cell line;ME180: Human cervical cancer cell line;MGMT: O6-methylguanine-DNA methyltransferase;MTT: Thiazolyl blue tetrazolium bromide;NBT-BCIP: Nitro blue tetrazolium- 5-Bromo-4-chloro-3-indolyl phosphate;NCI-H358: Human lung carcinoma cell line;NE-PER: Nuclear and Cytoplasmic Extraction Reagents;NF-κB: Nuclear factor type B;NLS: Nuclear signal localization;PANC-1: Human pancreatic cancer cell line;PBS: Phosphate buffered saline;PI3K/Akt: Phosphatidylinositol-3 kinases/ protein Kinase B;PTEN: Phosphatase and tensin homolog;SDS: Sodium dodecyl sulfate;SDS-PAGE: Sodium dodecyl sulfate polyacrylamide gel electrophoresis;SK-OV-3: Human ovarian carcinoma cell line;TMZ: Temozolomide;TransAM: DNA-binding ELISAs for activated NF-κB transcription factors;U87MG: Glioblastoma cell line;U937: Human leukemia cell line

## Competing interests

The authors declare that they have no competing interests.

## Authors’ contributions

MŻR - carried out the studies (MTT test), helped to draft the manuscript, helped to perform the statistical analysis. FA - carried out the Western blot and Elisa, participated in the H^3^-thymidine incorporation procedure, helped to draft the manuscript. NSK - carried out the studies (MTT test), helped to perform the statistical analysis, helped to draft the manuscript. SD - helped to perform the statistical analysis, and to draft the manuscript. CH – participated in design of the study, helped with the analysis and interpretation of data, helped to draft the manuscript. BMH - participated in design of the study, coordinated the study, helped with the analysis and interpretation of data helped to draft the manuscript. All authors read and approved the final manuscript.

## Pre-publication history

The pre-publication history for this paper can be accessed here:

http://www.biomedcentral.com/1472-6882/13/50/prepub
